# Dimeric γ-AApeptides With Potent and Selective Antibacterial Activity

**DOI:** 10.3389/fchem.2020.00441

**Published:** 2020-06-12

**Authors:** Minghui Wang, Ruixuan Gao, Peng Sang, Timothy Odom, Mengmeng Zheng, Yan Shi, Hai Xu, Chuanhai Cao, Jianfeng Cai

**Affiliations:** ^1^Department of Chemistry, University of South Florida, Tampa, FL, United States; ^2^College of Chemistry and Chemical Engineering, Central South University, Changsha, China; ^3^College of Pharmacy, University of South Florida, Tampa, FL, United States

**Keywords:** host defense peptide, γ-AApeptides, antimicrobial, Gram-positive strains, drug resistance

## Abstract

Over the past few decades, the emergence of antibiotic resistance developed by life-threatening bacteria has become increasingly prevalent. Thus, there is an urgent demand to develop novel antibiotics capable of mitigating this trend. Herein, we report a series of dimeric γ-AApeptide derivatives as potential antibiotic agents with limited toxicity and excellent selectivity against Gram-positive strains. Among them, compound **2** was identified to have the best MICs without inducing drug resistance, even after exposure to MRSA for 20 passages. Time-kill kinetics and mechanistic studies suggested that **2** could mimic host-defense peptides (HDPs) and rapidly eradicate MRSA within 2 hours through disturbing the bacteria membrane. Meanwhile, biofilm formation was successfully inhibited even at a low concentration. Taken together, these results suggested the great potential of dimeric γ-AApeptide derivatives as antibacterial agents.

## Introduction

The emergent antibiotic resistance has already limited the use of many antibiotics and has posed a great threat to public health (Shi et al., 2019a). In the United States, 2 million people get infected by antibiotic-resistant infections every year, which is estimated to cause more than 700,000 deaths (Nimmagadda et al., 2017; Wang et al., 2019). Over the last century Gram-positive bacteria, especially the notorious methicillin-resistant *Staphylococcus aureus* (MRSA), *Staphylococcus epidermid*is (MRSE), and *vancomycin-resistant E. faecal* (VRE), have spread easily and are now becoming the leading cause of bacterial infections within both health-care and community settings (Lee et al., 2018; Niu et al., 2018).

Considerable efforts have been extended to meet the urgent medical needs whereas the antibacterial drug discovery is experiencing a marked decrease (Payne et al., 2006). As an alternative strategy, host defense peptides (HDPs), also termed “antimicrobial peptides (AMPs),” are serving as a first-line defense against bacterial infections in eukaryotic organisms. (Méndez-Samperio, 2014; Teyssières et al., 2016) Previous reports have shown that HDPs, such as indolicidin (Galdiero et al., 2016) and human defensin 5 (Lei et al., 2018), are potential antibiotics based on their membrane disruption (permeabilization and lysis) ability. Unlike conventional antibiotics targeting some specific intracellular and cell wall targets, the main driving force in the selectivity is the electrostatic interaction between HDPs and bacterial membrane (Su et al., 2017; Niu et al., 2018). It is well known that the membrane of bacteria is negatively charged due to the presence of phosphatidylglycerol (PG), whereas mammalian cell membrane consists of zwitterionic sphingomyelin and phosphatidylcholine (PC). In addition, negatively charged lipopolysaccharides (LPS) and lipoteichoic acids (LTA) on the bacterial membrane further contribute to the selectivity of cationic HDPs for their recognition of bacteria cells (Teng et al., 2016a; Nimmagadda et al., 2017; Su et al., 2017; Niu et al., 2018). After initial selective contact with bacteria, lipophilic side chains of amphipathic HDPs could insert into the lipid bilayer to compromise the membrane stability and integrity through multiple modes, leading to eventual bacterial death (Niu et al., 2018). As the interaction is without the use of a specific protein or DNA targets, this mechanism could explain why HDPs have a low propensity to develop drug resistance, at least partially.

Although these advantages are promising, there are several drawbacks hindering HDPs to advance into clinical sittings, including low effectiveness, high toxicity, and difficulty during the synthesis.^12^ Recently, peptidomimetics that mimic HDPs have been developed in order to overcome the hurdles, such as α/β-peptides (Karlsson et al., 2006, 2010; Molchanova et al., 2017), α/β-peptoids (Chongsiriwatana et al., 2008, 2011), AApeptides (Shi et al., 2016; Teng et al., 2017b; Li et al., 2018), arylamide oligomers (Hua et al., 2010), and oligoacyllysines (Rotem et al., 2010). γ-AApeptides (oligomers of γ-substituted-N-acylated-N-aminoethyl amino acids), which have shown promising antimicrobial application, were developed based on the γ-PNA backbone (Teng et al., 2017a, 2018, 2019; She et al., 2018; Shi et al., 2019b). One of the most attractive advantages of γ-AApeptides is that the secondary amines on the backbone could be modified and thereby provide opportunities to introduce a wide variety of functional side chains. This could include the introduction of hydrophobic groups and cationic groups that can help mimic the HDPs (Hu et al., 2012; Wu et al., 2012; Li et al., 2014b; Teng et al., 2016b). Other advantages of γ-AApeptides are their higher bioavailability, resistance to proteolytic degradation, and the added benefit that they are easy to synthesize. We recently reported a series of antibacterial agents that are based on γ-AApeptides, which displayed good antibacterial activity (against both Gram-positive and Gram-negative bacteria) (Li et al., 2014a; Padhee et al., 2015; Teng et al., 2016a). Herein, we attempted to utilize a dimerization strategy while developing novel amphiphilic antibiotics based on small sized γ-AApeptides.

## Results and Discussion

### Design and Synthesis of Dimeric AApeptides

**Figure 1:**
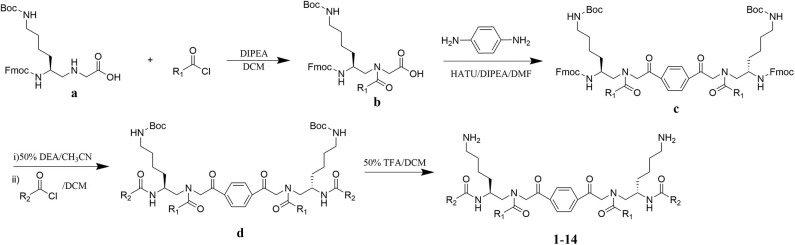
General synthesis of intermediates **(b–d)** and compounds **1–14**. Start material **(a)** has been reported before.

The design of dimeric AApeptides is a very straightforward concept through the use of solid phase peptide chemistry. In order to mimic an amphipathic HDPs bearing both a cationic and hydrophobic group, we prepared lysine-derived AApeptide building blocks containing variable hydrophobic side chains (R_1_) (Figure 1b) (Teng et al., 2016a; Su et al., 2017; Niu et al., 2018). Following that, a *p*-phenylenediamine was used as a linker to dimerize two building blocks (**b**) (Becker et al., 2001; Debnath et al., 2012; Ghosh and Brindisi, 2015). The effort led to a series of dimeric AApeptides after removal of the Fmoc groups, followed by capping with R_2_ tails and final TFA deprotection. Some structures without R_2_ tails were also synthesized to evaluate the relationship between the antibacterial activity and the number of cationic groups. We believe the straightforward synthesis of these AApeptides could enhance their potential as practical antibiotic agents.

### *In vitro* Minimum Inhibitory Concentration (MIC) Test and Hemolytic Assay

Having obtained all compounds, their antibacterial efficacy was subsequently evaluated by measuring the minimum inhibitory concentration (MIC). Three significant resistant bacteria, including methicillin-resistant *Staphylococcus aureus* (MRSA) (ATCC 33591), *Staphylococcus epidermid*is (MRSE) (RP62A), and *vancomycin-resistant E. faecalis* (VRE) (ATCC 700802), were chosen for our studies. Meanwhile, hemolytic activity (HC_50_) was also measured to assess the selectivity of these compounds toward bacteria and blood cells. To our delight, most of these compounds showed strong activity against all tested strains. As shown in Table 1, the most potent compound **2** exhibited MICs less than 10 μg/mL toward all evaluated bacteria, and the activity against MRSA is only 2 μg/mL. Importantly, HC_50_ of almost all compounds are equal or more than 250 μg/mL. This indicated that all active compounds are highly selective toward Gram-positive bacteria. The compounds also displayed decent structure activity relationship as expected. **2** and **3** showed excellent activity against both MRSA and MRSE while **2** has a twofold-lower MIC toward VRE. This result indicated that alkyl tails with 10 or 12 carbons were effective to eradicate bacteria cells. Interestingly, with the length of the alkyl tail increased to 14 and 16, compounds **4** and **5** showed activities against all strains over 25 μg/mL. This is consistent with our previous findings that significant hydrophobicity, particularly two long alkyl tails, could form hydrophobic clusters that prevent the formation of amphipathic structures necessary for antimicrobial activity (Li et al., 2014b; Singh et al., 2018). By contrast, decreasing carbon tails to 6 (compound **1**) greatly improved the activity against MRSE to 0.75 μg/mL, while losing the effectiveness toward MRSA. MICs of **10**–**14** unveiled that proper bulky hydrophobic groups were beneficial in the design of bactericide. These five compounds have the same (3r,5r,7r)-1-methyladamantane side chain but different tails, including C9, as well as C11 alkyl chain, ethylbenzene, and (3r,5r,7r)-1-methyladamantane. Among them, **10**, which had no tail, was the best toward MRSA and MRSE, with the MICs of 5 μg/mL and 2 μg/mL respectively. Interestingly, **11** and **14**, which had long fatty chains, did not show activity against any of bacterial strains tested up to 25 μg/mL, while **12** and **13** with bulky tails showed much better activity against all three strains. Compounds **7** and **8** also shared a similar trend. It appears that compounds with bulky groups have better activity, due to the lager molecular volume or shorter molecular length of these bulky groups. Therefore, the selection of proper hydrophobic bulky groups when designing HDP-mimicking agents is considerably crucial (Wu et al., 2012; Teng et al., 2016a).

**Table 1:** Structures, MICs, and HC_50_ of compound **1**-**14**^a^.

**#**	**Structure**	**MIC (μg/mL)**			**Hemolysis** **(HC_**50**_, μg/mL)**	**Selectivity index (HC_**50**_/MIC of MRSA)**
		**MRSA**	**MRSE**	***VRE***		
**1**	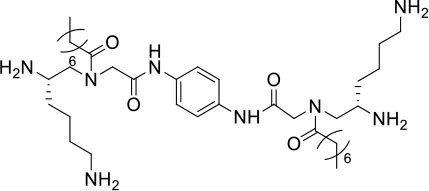	2	<0.75	>25	>250	>125
**2**	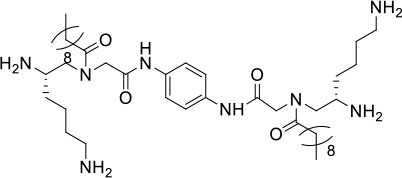	2	1	10	250	125
**3**	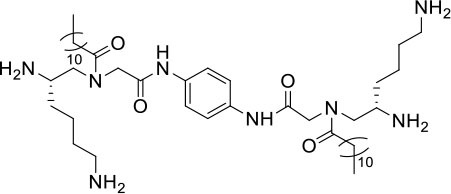	2	1	20	250	>125
**4**	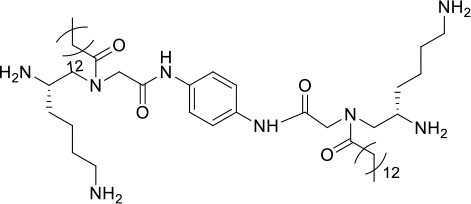	>25	>25	>25	>250	-
**5**	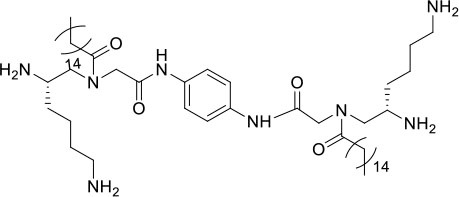	>25	>25	>25	>250	-
**6**	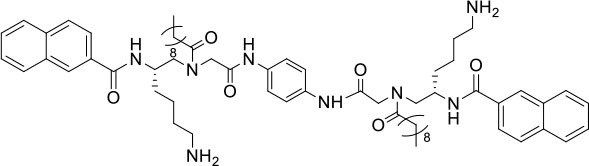	20	>25	>25	>250	>12.5
**7**	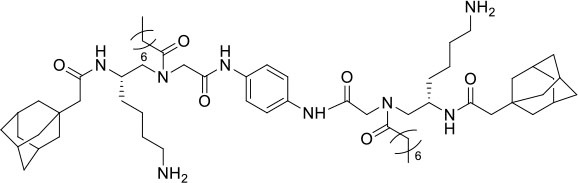	10	2	20	250	25
**8**	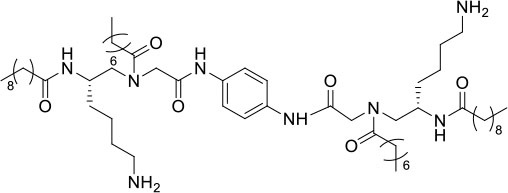	>25	>25	>25	250	-
**9**	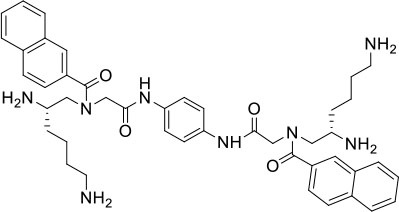	2	10	>25	>250	>125
**10**	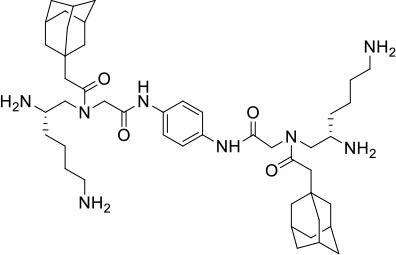	5	1	20	>250	>50
**11**	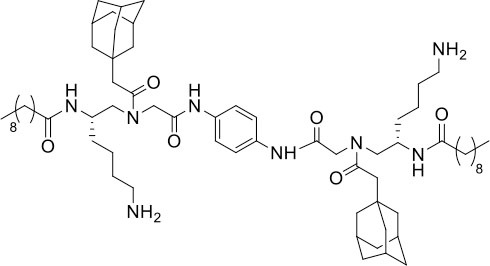	>25	>25	>25	>250	-
**12**	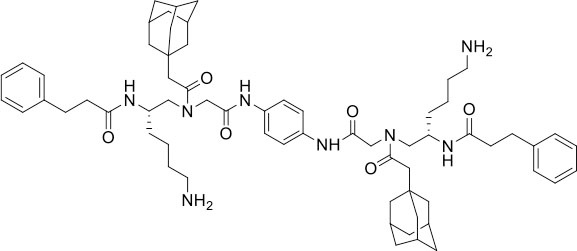	5	2	10	125	25
**13**	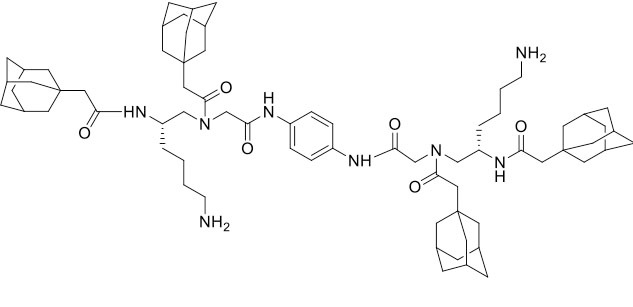	5	2	20	250	50
**14**	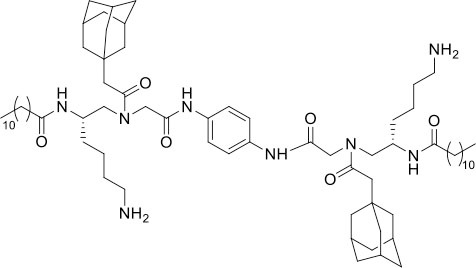	>25	>25	>25	>250	-

a *Bacteria included in the test were methicillin-resistant S. aureus (MRSA) (ATCC 33591), methicillin-resistant S. epidermidis (MRSE) (RP62A), and vancomycin-resistant E. faecalis (ATCC 700802). The most potent compound **2** is shown in red. The experiments were repeated three times*.

### Bactericidal Time-Kill Kinetics

**Figure 2:**
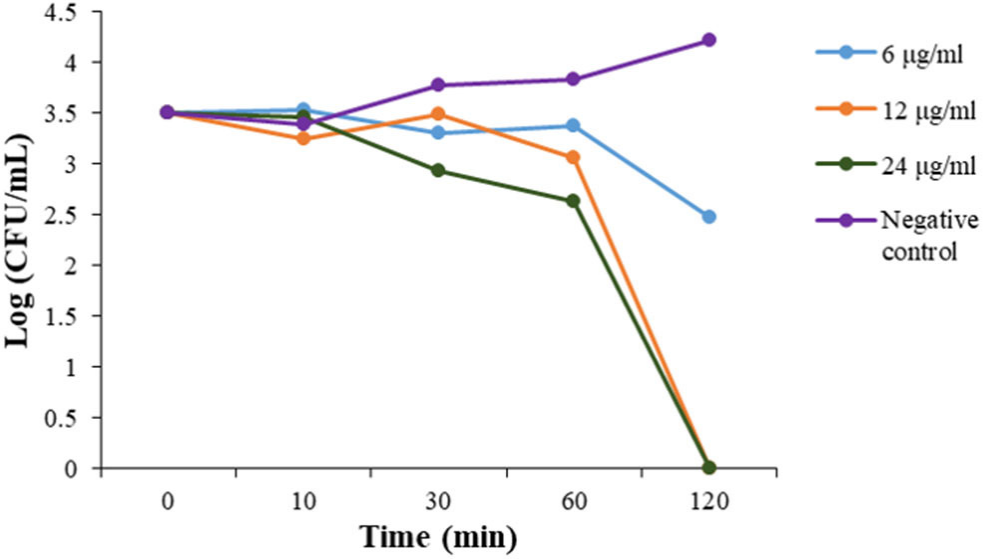
Time-kill kinetics of **2** against MRSA. The experiments were repeated three times.

To evaluate the action mode of our synthetic antibacterial agents, the kinetics of MRSA killing were conducted for the most potent compound **2** at the concentrations of 6 μg/mL, 12 μg/mL, and 24 μg/mL respectively. As shown in Figure 2, at the concentrations of 12 μg/mL and 24 μg/mL, all bacteria could be eradicated within 120 mins. Although there were still colonies after 120 mins with treatment of **2** at the 6 μg/mL, the growth of bacteria had an obvious decreasing trend, which indicated that bacteria may all have been eradicated after a longer treatment. The data indicate that compound **2** could eradicate MRSA rapidly.

### Bacteria Membrane Depolarization

**Figure 3:**
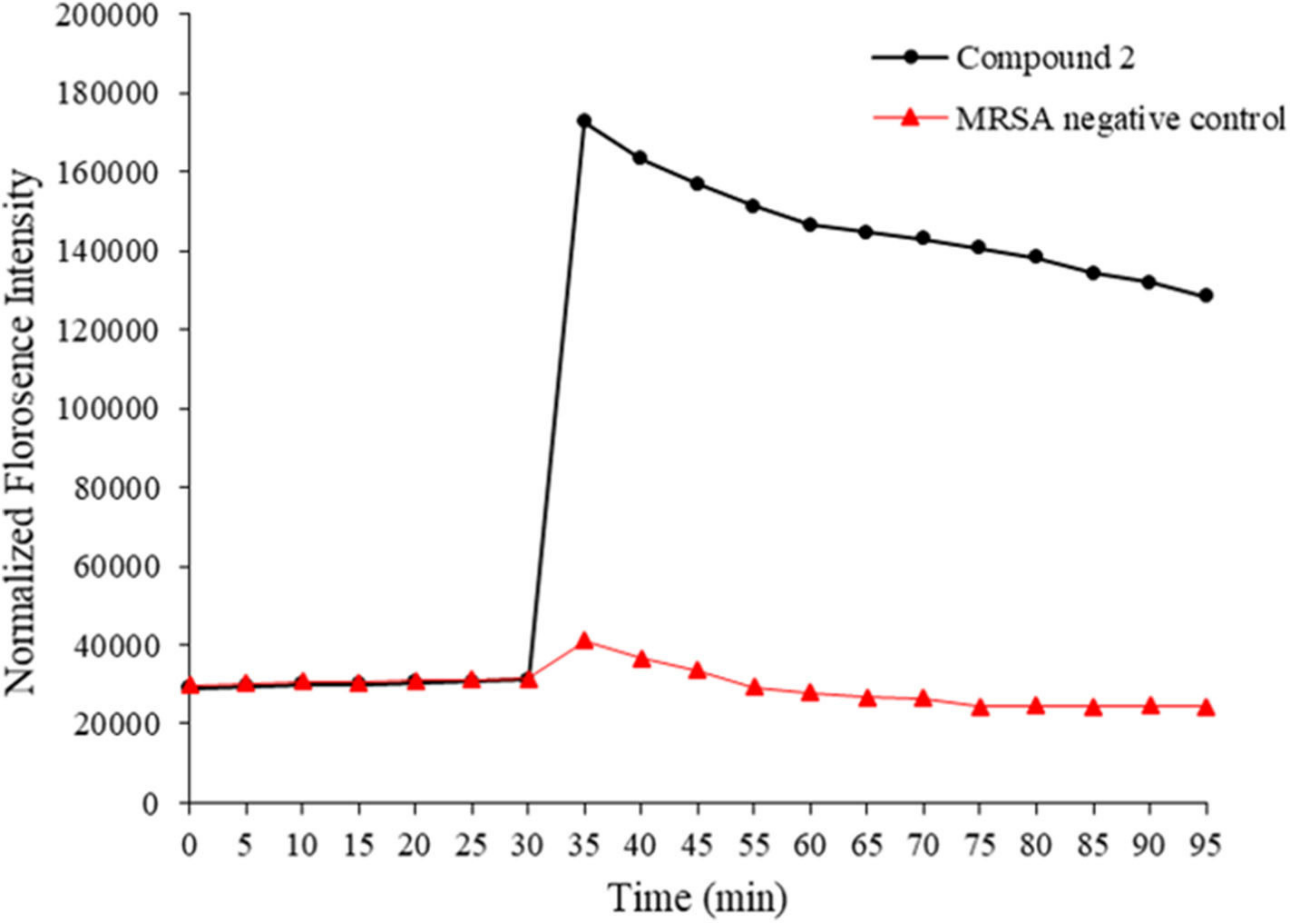
Membrane depolarization against MRSA. Negative control is bacteria without treatment of antibacterial agents. The experiments were repeated three times.

HDPs are known to act on the bacterial membrane. As the mimics of HDPs, compounds **1–14** were also expected to kill bacteria through disintegrating the bacterial cell membrane. To this end, 3, 3′- Dipropylthiadicarbocyanine iodide [DiSC3(5)] was used as a fluorogenic probe to detect and measure changes in transmembrane potential. Briefly, fluorescence intensity is very weak when it accumulates on hyperpolarized membranes due to their self-quenching (Niu et al., 2018). However, as long as the integrity of the membrane is interrupted, the fluorescence intensity would increase significantly. This method was used to initially determine the bacterial membrane permeability caused by the compound **2**. As shown in Figure 3, the fluorescence intensity has no big difference between two groups within 30 mins. However, after the treatment of MRSA with compound **2**, the fluorescence intensity increased dramatically, whereas the negative control had no big difference. The result suggested that compound **2** disintegrated the MRSA cell membrane, which is the typical antibacterial mode of HDPs.

### Fluorescence Microscopy

**Figure 4:**
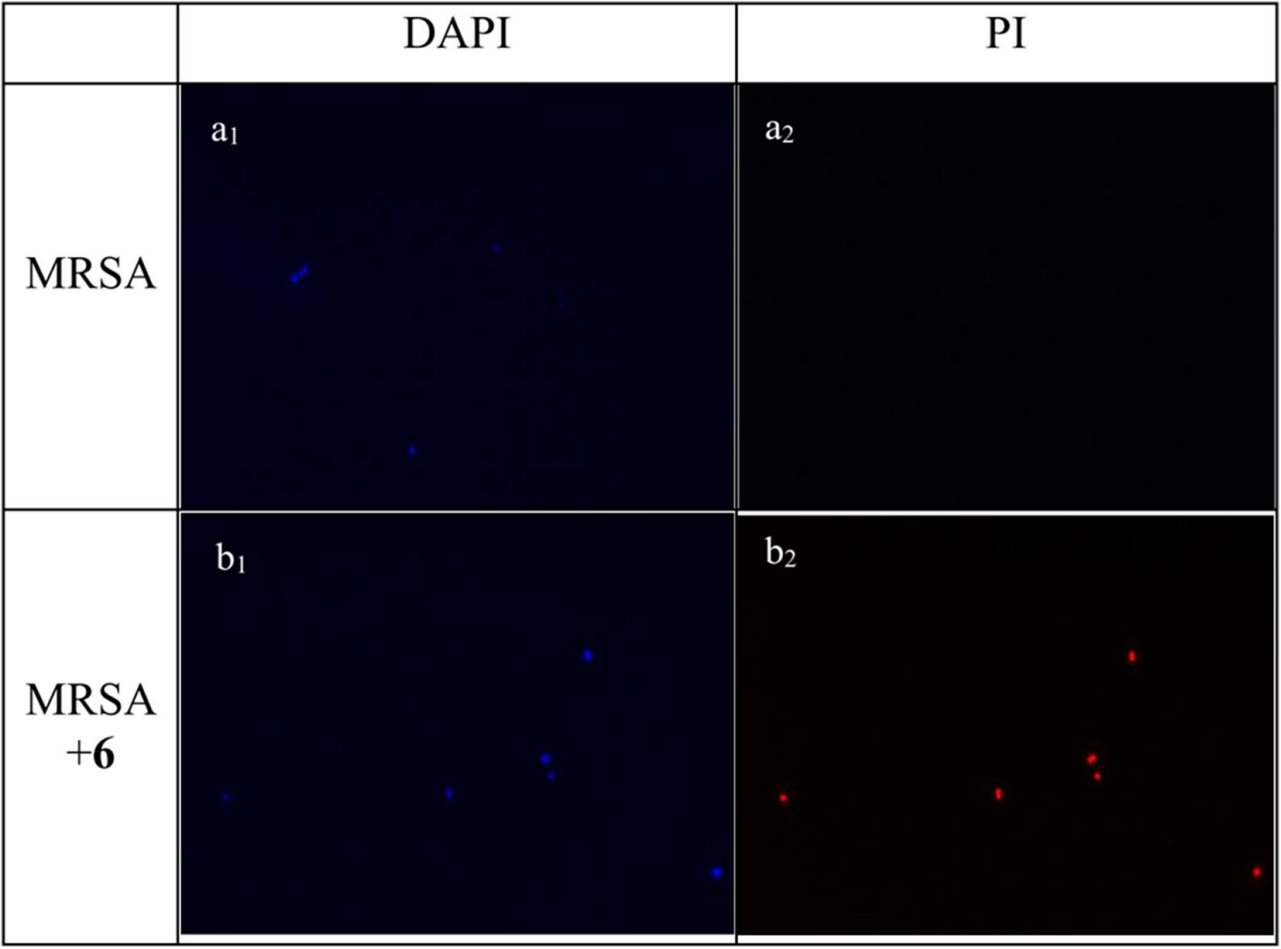
Fluorescence micrographs of MRSA in DAPI and PI channels. Bacteria was observed under 40 folds lens. **(a**_**1**_**)** Control, no treatment, DAPI channel. **(a**_**2**_**)** Control, no treatment, PI channel. **(b**_**1**_**)** MRSA treated with compound **2**, DAPI channel. **(b**_**2**_**)** MRSA treated with compound **2**, PI channel. The experiments were repeated three times.

Experiments of fluorescent microscopy is also a broadly used method to determine the integrity of bacteria membrane. Propidium iodide (PI) and 4′,6-diamidino-2-henylindole (DAPI) are commercially available dyes that stain dead or injured cells with compromised membrane and all cells irrespective of their viability, respectively. The efficacy of compound **2** to interrupt the bacterial membrane was evaluated by these two dyes. As shown in Figure 4, after being stained by DAPI and PI respectively, MRSA negative control only has a bright blue fluorescent in the DAPI channel, which demonstrated that only DAPI was stained on bacterial membrane, suggesting the membrane was intact. When MRSA was treated with compound **2** at the concentration of 6 μg/mL for 2 h, fluorescence could be detected in both channels, indicating that the membrane of MRSA was interrupted after the treatment of **2**.

### TEM Study

**Figure 5:**
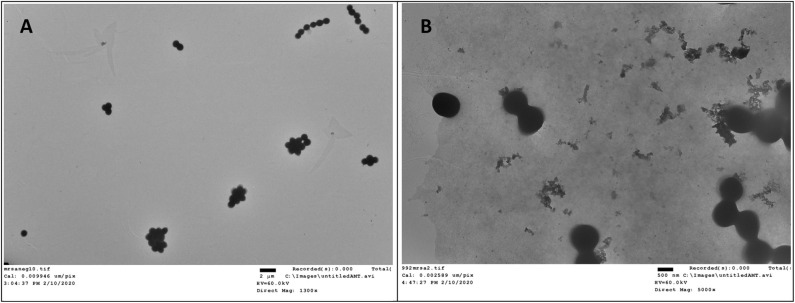
TEM study of compound **2** against MRSA. **(A)** Control, no treatment. **(B)** MRSA treated with compound **2**. The experiments were repeated three times.

Transmission electron microscopy (TEM) is a straightforward method to visualize the interruptive effect of compounds on the membrane of bacteria. Mid log phase MRSA without the treatment of compound **2** had intact membrane (Figure 5A). After incubation with **2** at the concentration of 6 μg/mL for 2 h, large amount of cell debris can be detected, demonstrating that the membrane was disintegrated (Figure 5B).

### Bacteria Resistance Study

**Figure 6:**
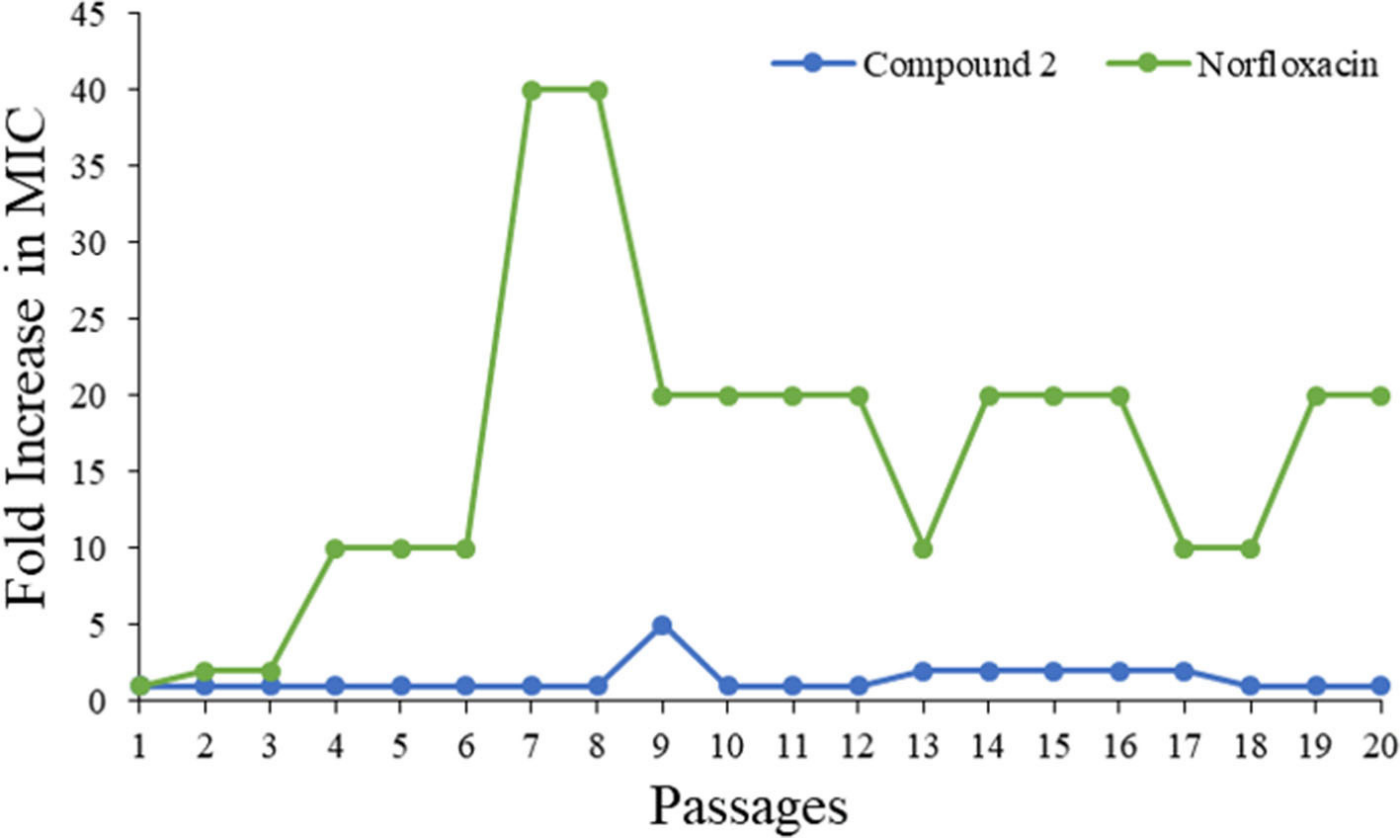
Drug resistance of compound **2** and Norfloxacin against MRSA.

Drug resistance has already been recognized as one of the fastest growing threats all over the world. As such, a bacteria resistance study was carried out to detect whether the most potent compound **2** could be a prospective compound to overcome the issue. The MIC of compound **2** was determined daily, and then the bacteria suspension with half of the MIC was diluted to 10^6^ CFU/mL and incubated with compound to test its activity until 20 passages had been reached. As shown in Figure 6, the MIC values of compound **2** were almost unchanged even after 20 passages, while that of norfloxacin increased 10-fold. This result demonstrated that **2** had minimum propensity to induce bacterial resistance.

### Inhibition of Biofilm

**Figure 7:**
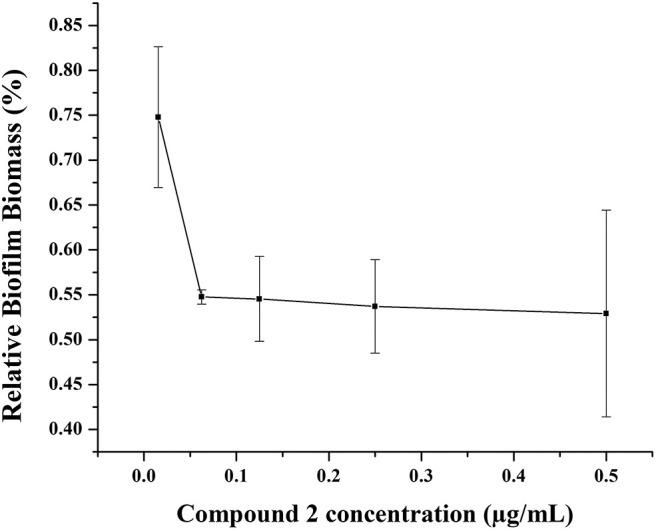
Biofilm disruption of compound **2**.

Biofilms, which have been increasingly recognized as a main accessory of drug resistance, is a community of bacteria living in cellular cluster or microcolonies encapsulated in a matrix consisted of extracellular polymers (Davies, 2003). Bacteria in biofilm are 10- to 1,000-fold more resistance to antibacterial agents (Hoque et al., 2015). Therefore, it is very significant to develop antibiotics with potency not only to inhibit the growth of biofilm, but also to eradicate the formed biofilm. Herein, one of our best compounds, **2**, was evaluated the eradication of established MRSA biofilm. As shown in Figure 7, at a concentration of 0.06 μg/mL, 55% biofilm can be inhibited by compound **2**. This result indicated the effectiveness of compound **2** as an antibacterial agent and was consistent with its satisfactory anti-drug resistance activity.

## Conclusion

In summary, a series of novel dimerized γ-AApeptide antibacterial compounds, mimicking HDPs, were rationally designed and synthesized. Most of these compounds showed excellent bacteria-killing efficacy against a panel of medicinally relevant multidrug resistant Gram-positive bacteria including MRSA, MRSE, and VRE. Notably, these compounds also exhibited high specificity toward bacteria, suggesting they are safe and promising agents to be applied for clinical use. Mechanism studies of the most potent compound **2** demonstrated that the compounds kill bacteria rapidly by interrupting membrane integrity, a way analogous to HDPs. Furthermore, compound **2** did not induce drug resistance, augmenting its therapeutic potential. Together with their small size and straightforward synthesis, the dimerized γ-AApeptides could be developed into a new generation of potential antibacterial agents to combat drug resistance.

## Experimental Section

### General Information

All chemicals and solvent were purchased from either Sigma-Aldrich or Fisher Scientific and were used without further purification. All reactions were monitored by thin layer chromatography. Column chromatography was carried out with silica gel (200–300 mesh). Visualization was accomplished by using a UV (254 nm) lamp. The final products were purified on a Waters Breeze 2 HPLC system and lyophilized on a Labconco lyophilizer. The purity of the compounds was determined to be >95% by analytical HPLC [1 mL/min flow, 5–100% linear gradient of solvent B (0.1% TFA in acetonitrile) in A (0.1% TFA in water] over 40 min was used (detection wavelength was 215 nm or 254 nm). High resolution mass spectra of compounds were identified by an Agilent Technologies 6540 UHD accurate-mass Q-TOF LC/MS spectrometer. TEM images were obtained on a FEI Morgagni 268D TEM with an Olympus MegaView III camera on the microscope.

### Synthesis and Characterization

#### N,N'-(((1,4-phenylenebis(2-oxoethane-2,1-diyl)) bis ((2-((3r,5r,7r)-adamantan-1-yl) acetyl) azanediyl)) bis (6-aminohexane-1,2-diyl)) bis (decanamide) (11)

The synthesis of other compounds is similar to **11**. The synthesis of structure **a** followed the method reported before (Teng et al., 2016b). 2-((3r,5r,7r)-adamantan-1-yl)acetyl chloride (625 μL, 2.94 mmol) was added dropwise into the DCM (20 mL) solution of **a** (500 mg, 0.98 mmol) and diisopropylethylamine (DIPEA) (512 μL, 2.94 mmol). After stirring for 2 h at room temperature, the solution was diluted with 10 mL DCM and then washed with 1 M HCl (15mL × 1), brine (15mL × 1), and then dried with Na_2_SO_4_ to afford crude **b**. After the solvent was removed under reduced pressure, the residue was purified by column chromatography on silica gel (petroleum ether: ethyl acetate = 1:1) to afford the pure product **b** (619 mg, 92%). 1-[Bis(dimethylamino)methylene]-1H-1,2,3-triazolo[4,5-b]pyridinium 3-oxid hexafluorophosphate (HATU) (441 mg, 1.16 mmol) was added to the DMF (20 mL) solution of 200 mg (0.29 mmol) **b**. Then, DIPEA (202 μL, 1.16 mmol) was dropped into the above solution while stirring. At last, benzene-1,4-diamine (15.6 mg, 0.15 mmol) was added into the solution. After 2 h, the mixture was diluted with ethyl acetate 50 mL and then washed with 15 ml 1 M HCl, 50 mL × 3 brine. Na_2_SO_4_ was applied to dry the solution. After being concentrated by rotavapor, crude **c** (169 mg, 83%) was obtained and used without further purification. CH_3_CN:ethylenediamine = 1:1 solution was added to **c** to stir 30 min. After the solution was removed, cold ether was used to precipitate the crude product and the residue was blown to dry. Decanoyl chloride (91μL, 0.48 mmol) was dropped into the CH_2_Cl_2_ (20 mL) solution with the crude product obtained from last step and DIPEA (84 μL, 2.3 mmol). After stirring for 2 h, the solution was washed with 1 M HCl (15mL × 1) and brine and dried with Na_2_SO_4_ to afford crude **d**. Then, after evaporating the solution without any purification, CH_2_Cl_2_:trifluoroacetic acid = 1:1 was added into a flask with **c**, stirring at room temperature for 1 h. The whole solution was removed by rotavapor after the reactant had been completely consumed. Crude final product **11** was dissolved by CH_3_CN:H_2_O = 2:1 and then purified by HPLC to obtain the pure compound **11** (26 mg, 20%) as a white solid. HRMS (ESI-TOF) m/z C_52_H_82_O_8_N_6_ [M+H]^+^ calcd= 1111.8627; found= 1111.8670.

For compounds **1**–**5** and **9**–**10**, after obtaining the intermediate **c**, CH_3_CN:ethylenediamine = 1:1 and DCM:TFA = 1:1 was directly used to remove Fmoc protecting group and Boc protecting group following the method introduced above. All trace and Q-tof spectrum information is included in Support Information.

*N,N'*-((1,4-phenylenebis(azanediyl))bis(2-oxoethane-2,1-diyl))bis(N-(2,6-diaminohexyl) octanamide) (**1**) HRMS (ESI-TOF) m/z C_38_H_70_O_8_N_4_ [M+H]^+^ calcd = 703.5598; found = 703.5592.

*N,N'*-((1,4-phenylenebis(azanediyl))bis(2-oxoethane-2,1-diyl))bis(N-(2,6-diaminohexyl) decanamide) (**2**) HRMS (ESI-TOF) m/z C_42_H_78_O_8_N_4_ [M+H]^+^ calcd = 759.6224; found = 759.6243.

*N,N'-*((1,4-phenylenebis(azanediyl)) bis (2-oxoethane-2,1-diyl)) bis (N-(2,6-diaminohexyl) dodecanamide) (**3**) HRMS (ESI-TOF) m/z C_46_H_86_O_8_N_4_ [M+H]^+^ calcd = 815.6850; found= 815.6880.

N,N'-((1,4-phenylenebis(azanediyl))bis(2-oxoethane-2,1-diyl))bis(N-(2,6-diaminohexyl) tetradecanamide) (**4**) HRMS (MALDI-TOF) m/z C_50_H_94_N_8_O_4_ [M+H]^+^ calcd = 871.7476; found= 871.9586.

N,N'-((1,4-phenylenebis(azanediyl))bis(2-oxoethane-2,1-diyl))bis(N-(2,6-diaminohexyl) palmitamide) (**5**) HRMS (MALDI-TOF) m/z C_54_H_103_N_8_O_4_ [M+H]^+^ calcd = 928.4700; found = 928.1901.

*N,N'*-((((1,4-phenylenebis(azanediyl))bis(2-oxoethane-2,1-diyl)) bis (decanoylazanediyl)) bis(6-aminohexane-1,2-diyl))bis(2-naphthamide) (**6**) HRMS (ESI-TOF) m/z C_64_H_90_O_8_N_6_ [M+H]^+^ calcd = 1,067.7062; found = 1,067.7017.

*N-*(2-(2-((1s,3s)-adamantan-1-yl)acetamido)-6-aminohexyl)-N-(2-((4-(2-(N-(2-(2-((3r,5r,7r)-adamantan-1-yl)acetamido)-6-aminohexyl) decanamido) acetamido) phenyl) amin)−2-oxoethyl)decanamide (**7**) HRMS (ESI-TOF) m/z C_62_H_103_O_8_N_6_ [M+H]^+^ calcd = 1,055.8001; found = 1,055.7969.

*N,N'-*((((1,4-phenylenebis(azanediyl))bis(2-oxoethane-2,1-diyl))bis(octanoylazanediyl)) bis(6-aminohexane-1,2-diyl))bis(decanamide) (**8**) HRMS (ESI-TOF) m/z C_58_H_106_O_8_N_6_ [M+H]^+^ calcd = 1,011.8314; found = 1,011.8356.

*N,N'-*((1,4-phenylenebis(azanediyl))bis(2-oxoethane-2,1-diyl))bis(N-(2,6-diaminohexyl)-2-naphthamide) (**9**) HRMS (ESI-TOF) m/z C_44_H_54_O_8_N_4_ [M+H]^+^ calcd = 759.4346; found = 759.4323.

*N,N'*-((1,4-phenylenebis(azanediyl))bis(2-oxoethane-2,1-diyl))bis(2-((3r,5r,7r)-adamantan-1-yl)-N-(2,6-diaminohexyl) acetamide) (**10**) HRMS (ESI-TOF) m/z C_46_H_74_O_8_N_4_ [M+H]^+^ calcd = 803.5911; found = 803.5888.

*N,N'*-((((1,4-phenylenebis(azanediyl))bis(2-oxoethane-2,1-diyl)) bis ((2-((3r,5r,7r)-adamantan-1-yl)acetyl)azanediyl))bis(6-aminohexane-1,2-diyl))bis(decanamide) (**11**) HRMS (ESI-TOF) m/z C_66_H_110_N_8_O_6_ [M+H]^+^ = 1,111.8627; found = 1,111.8670.

*N,N'*-((((1,4-phenylenebis(azanediyl)) bis (2-oxoethane-2,1-diyl)) bis ((2-((3r,5r,7r)-adamantan-1-yl)acetyl)azanediyl))bis(6-aminohexane-1,2-diyl)) bis (3-phenylpropanamide) (**12**) HRMS (ESI-TOF) m/z C_64_H_90_O_8_N_6_ [M+H]^+^ calcd = 1,067.7064; found = 1,067.7030.

*N,N'-*((1,4-phenylenebis(azanediyl))bis(2-oxoethane-2,1-diyl))bis(2-((3r,5r,7r)-adamantan-1-yl) -N-(2-(2-((3r,5r,7r) -adamantan-1-yl) acetamido)−6-aminohexyl) acetamide) (**13**) HRMS (ESI-TOF) m/z C_58_H_106_O_8_N_6_ [M+H]^+^ calcd = 1,155.8314; found= 1,155.8282.

*N,N'*-((((1,4-phenylenebis(azanediyl))bis(2-oxoethane-2,1-diyl))bis((2-((3r,5r,7r)-adamantan-1-yl)acetyl)azanediyl))bis(6-aminohexane-1,2-diyl))didodecanamide (**14**) HRMS (ESI-TOF) m/z C_58_H_106_O_8_N_6_ [M+H]^+^ calcd = 1,167.9253; found = 1,167.9215.

### Minimum Inhibitory Concentrations (MICs) against Bacteria

The antimicrobial assay of the compounds was conducted on the following three bacteria strains: *methicillin-resistant S.aureus* (MRSA, ATCC 33591), *methicillin-resistant S. epidermidis* (MRSE, RP62A), and *vancomycin-resistant E. faecalis* (VRE, ATCC 700802). The procedures were followed as reported previously. (Isaksson et al., 2011) The MICs were determined as the lowest concentration that completely inhibits the bacteria growth. All measurements were repeated at least three times with duplicates each time.

### Hemolysis Study

Fresh red blood cell (RBC) of mice was collected by centrifuge with the speed of 500 *g* for 10 mins until the supernatant is clear. Then, the RBC was diluted with PBS for 20-fold. After adding 50 μL PBS in each vial on 96 plates, 1 mg/mL compounds were serial diluted until reaching the lowest concentration 1.9 μg/mL. Diluted RBC was added into each vial and incubated at 37°C for 1 h. Then, plates were centrifuged at 2,301 *g* (3,500 rpm) for 10 mins. 30 *uL* supernatant in each vial was added into 100 μL PBS and read Biotek Synergy HT plate reader at 540 nm. The hemolytic activity was calculated by the formula % hemolysis = (Abssample-AbsPBS) / (AbsTriton-AbsPBS) × 100%. 1% Triton X-100 were used as the positive control and 1 × PBS buffer was used as the negative control.

### Time-Kill Study

Time-kill kinetic study of compound **2** were investigated. After being incubated from one colony in 4 mL TSB medium overnight at 37 °C while shaking, MRSA was diluted 2,500 times to another fresh 4 mL TSB solution and grew for 6~8 h to mid-logarithmic phase. Suspension [10^6^ colony-forming units per milliliter (CFU/mL) according to the read of OD value] was made and then incubated with different concentration of **2** at 37°C for 10 min, 30 min, 60 min, 120 min respectively. The MRSA and drug mixture were diluted 10^2^-fold and spread on the TSB agar plates. These plates were incubated at 37°C overnight. The numbers of colonies on each plate were transferred to lg[CFU/mL] and plotted against the incubation time. The experiment was repeated three times with duplicates each time.

### Fluorescence Microscopy

Fluorescent dyes propidium iodide (PI) and 4′,6′-diamidino-2-phenylindole dihydrochloride (DAPI) can be used to distinguish the viable cell from the dead cells. After obtaining the mid-logarithmic phase bacteria solution as above, the bacteria solution was diluted 100 times and mixed with compound **2** at the concentration of 6 μg/mL. Then the mixture was incubated 2 h and centrifuged at 1,690 *g* (3,000 rpm) for 15 min at 4°C to harvest the cells. After washing with PBS for 3 times, PI (5 μg/mL) and DAPI (10 μg/mL) was used to dye cells 20 min respectively. Controls were carried out without compounds. The bacterial cells were then visualized and analyzed by employing the LEICA DM 2000 optical microscope with an oil-immersion objective (40 ×). The experiment was repeated three times with duplicates each time.

### Membrane Depolarization Study

Mid log phase MRSA bacteria solution was obtained as above. Then, cells were collected and washed with 5 mM HEPES and 5 mM glucose respectively. 5 mM HEPES:5 mM glucose:100 mM KCl = 1:1:1 was used to resuspended bacteria cells (10^6^ CFU/mL). Following that, 200 μL of bacterial suspension and 1 μM DiSC3(5) were included in a 96-well plate, and the fluorescence of the suspension was monitored at room temperature for 30 min at excitation wavelength of 622 nm and emission wavelength of 670 nm. After 30 min, compound **2** was added to the wells and the increased potential was monitored. The experiment was repeated three times with duplicates each time.

### Drug Resistance Study

The experiment was carried out by following our previously reported protocol (Su et al., 2017; Niu et al., 2018). The study was conducted for 20 passages.

### TEM Study

After being incubated to mid log phase, MRSA(10^6^ CFU/mL) was mixed with 6 μg/mL compound **2** for 2 h. Then the bacteria cells were collected and washed with PBS and DI water. Following that, bacteria was resuspended in deionized water. Control bacterial samples were obtained without adding drugs. Above samples were applied to TEM grids by 5 μL bacteria solution, and the grids were allowed to dry in vacuum oven at the temperature of 45°C for 45 seconds. After being dried, TEM images were obtained on a FEI Morgagni 268D TEM with an Olympus MegaView III camera at 60 kV on the microscope. The microscope used AnalySiS software to run the camera.

### Biofilm Inhibition Study

Incubating the compound **2** of different concentrations and suspended bacteria (10^6^ CFU/mL) in TSB (Tryptic soy broth) medium at 37°C for 48 h. Then, the 96-well plate was reversed and the suspending bacteria was discarded. After washing with DI water for several times, biofilm was dried in air at room temperature. Subsequently, 125 μL 0.1% crystal violet was added into all vials to dye for 15 mins. Extra dye was discarded and washed. Adding 200 μL 30% acetic acid to dissolve colored biofilm. Finally, 125 μL acetic acid was transfer into another clean plate. OD values were read at 595 nm.

## Data Availability Statement

The raw data supporting the conclusions of this article will be made available by the authors, without undue reservation, to any qualified researcher.

## Ethics Statement

The animal study was reviewed and approved by the ethics committee of the University of South Florida (protocol number R IS00005111).

## Author Contributions

All authors listed made a substantial, direct and intellectual contribution to the work. MW synthesized all the compounds and conducted all the antibacterial assays. RG evaluated the hemolytic assay. PS provided valuable advice for compounds design. TO corrected the grammar mistakes and typos in this article, and he also helped with the experimental design and did the HPLC trace analysis. MZ finished the Q-tof spectrum test. YS provided suggestions on synthesis. HX provided methods on antibacterial assays. CC provided biolevel II lab and mice blood. JC designed all structures and provided funding.

## Conflict of Interest

The authors declare that the research was conducted in the absence of any commercial or financial relationships that could be construed as a potential conflict of interest.
