# Supplementary material for: Dimeric γ-AApeptides With Potent and Selective Antibacterial Activity
Source: Front Chem. 2020 Jun 12;8:441. doi: 10.3389/fchem.2020.00441 (PMC7304243; doi:10.3389/fchem.2020.00441)
Supplement: Supplementary file 1 [file Data_Sheet_1.pdf]

Support information

Dimeric γ-AApeptides with potent and selective antibacterial activity

Minghui Wang,[1] Ruixuan Gao, [1] Peng Sang, [1] Timothy Odom, [1] Mengmeng Zheng, [1] Yan Shi, [1] Hai Xu,[2] Chuanhai Cao,[3] and Jianfeng Cai*,[1]

[1]Department of Chemistry, University of South Florida, Tampa, FL, USA

[2]College of Chemistry and Chemical Engineering, Central South University, Changsha, Hunan, China

[3]College of Pharmacy, University of South Florida, Tampa, FL, USA.

HPLC and HRMS spectra of compounds 1−14

Compound 1

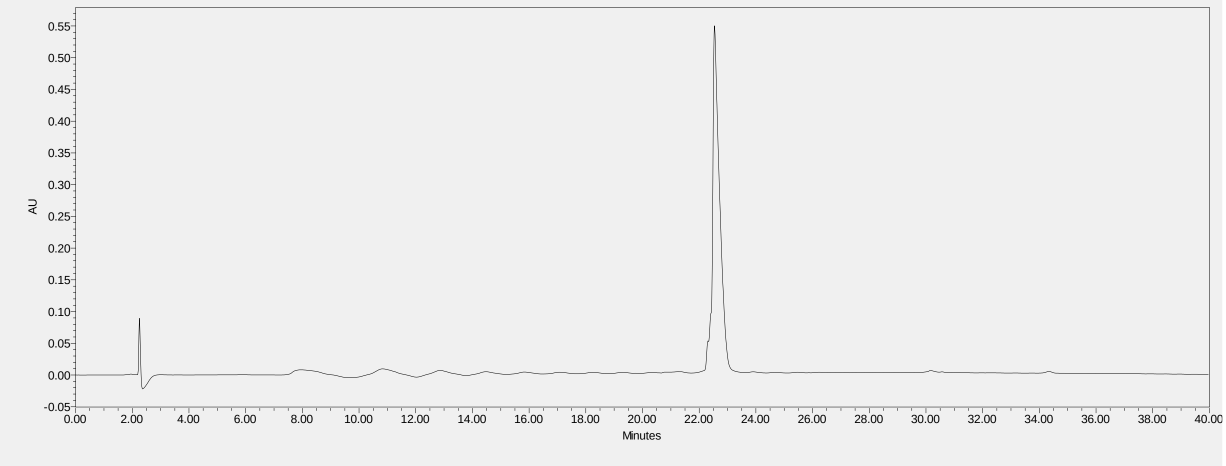

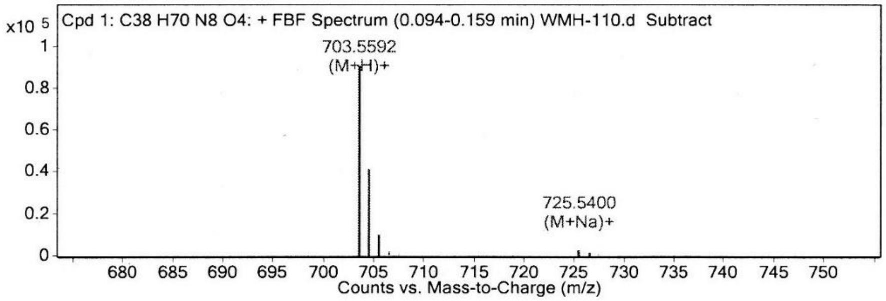

Compound 2

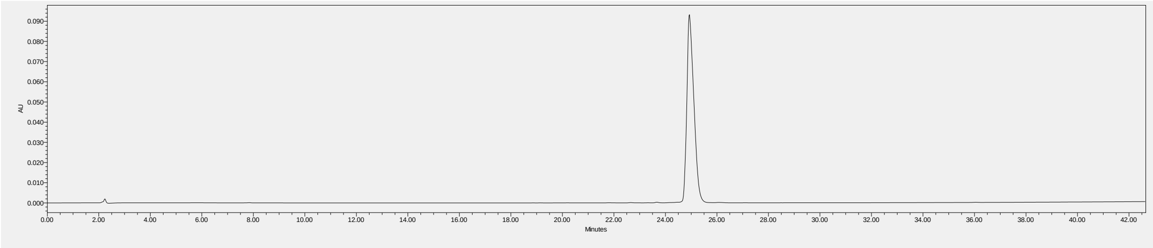

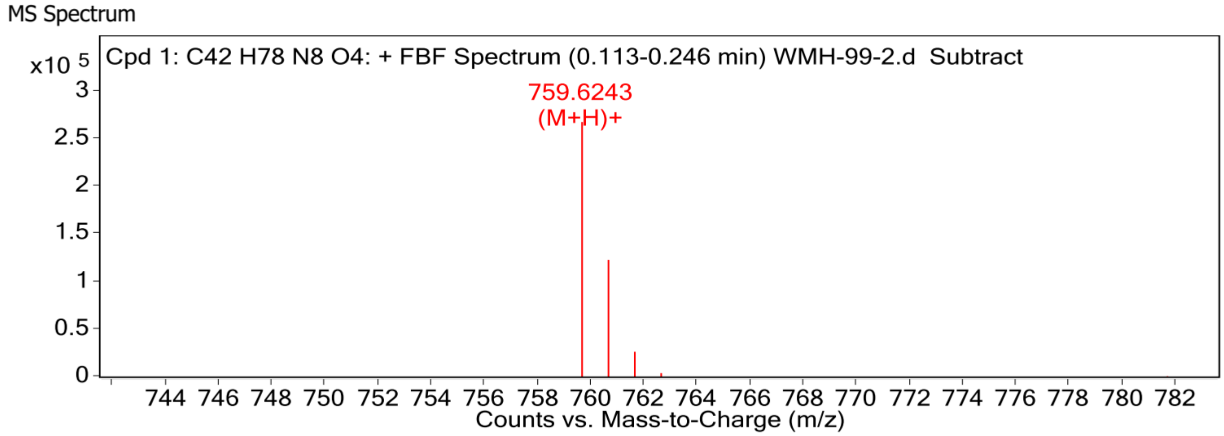

Compound 3

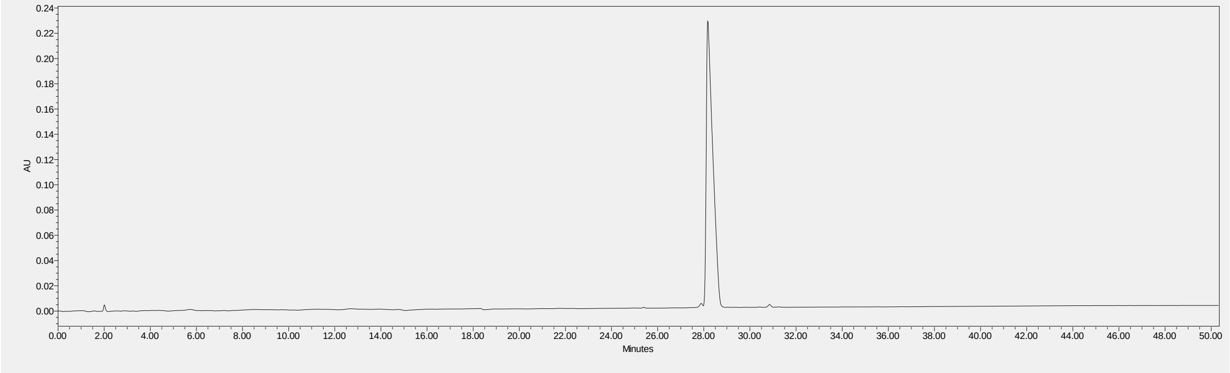

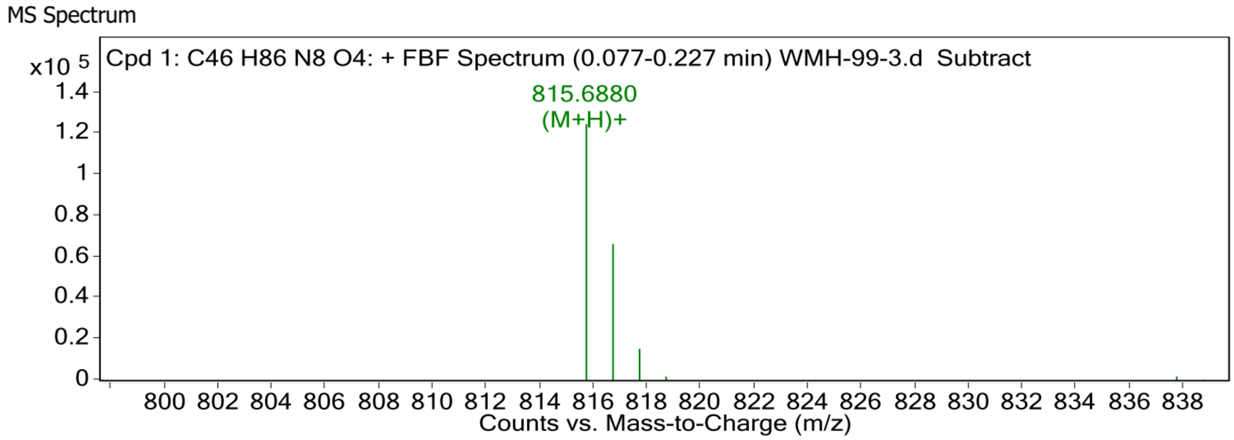

Compound 4

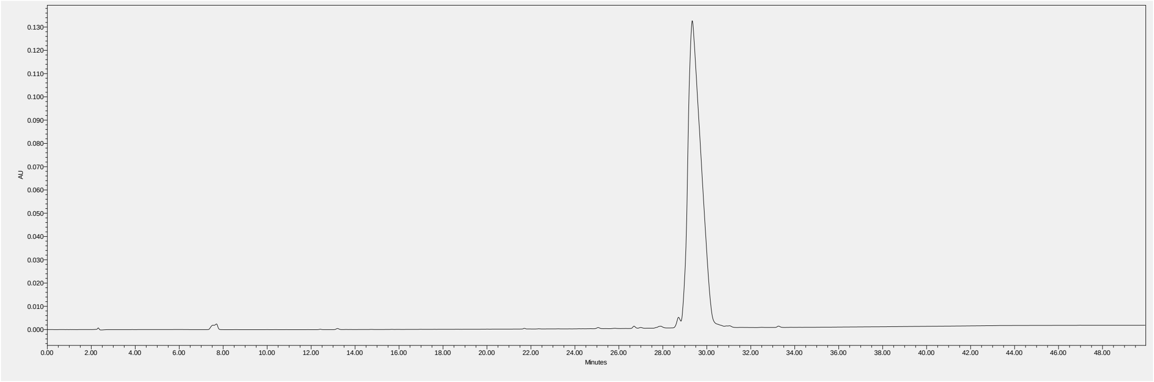

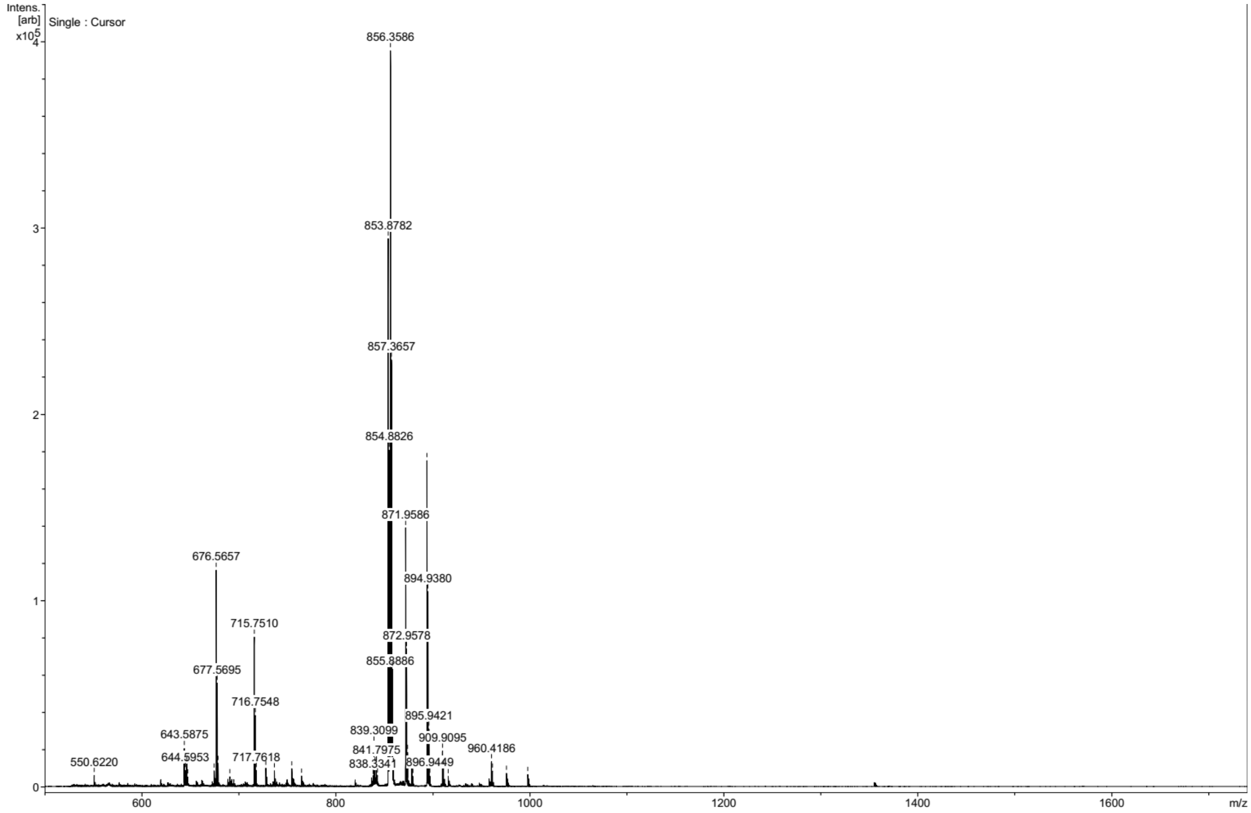

Compound 5

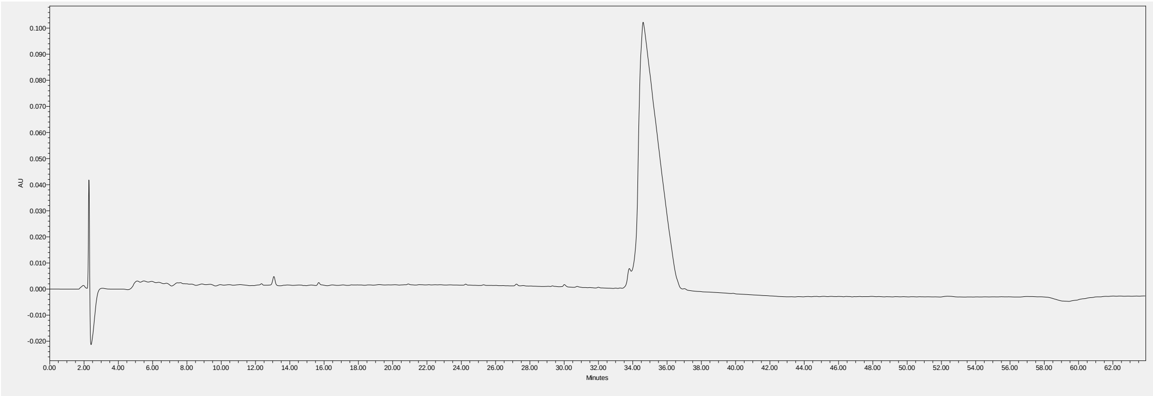

AU
Minutes

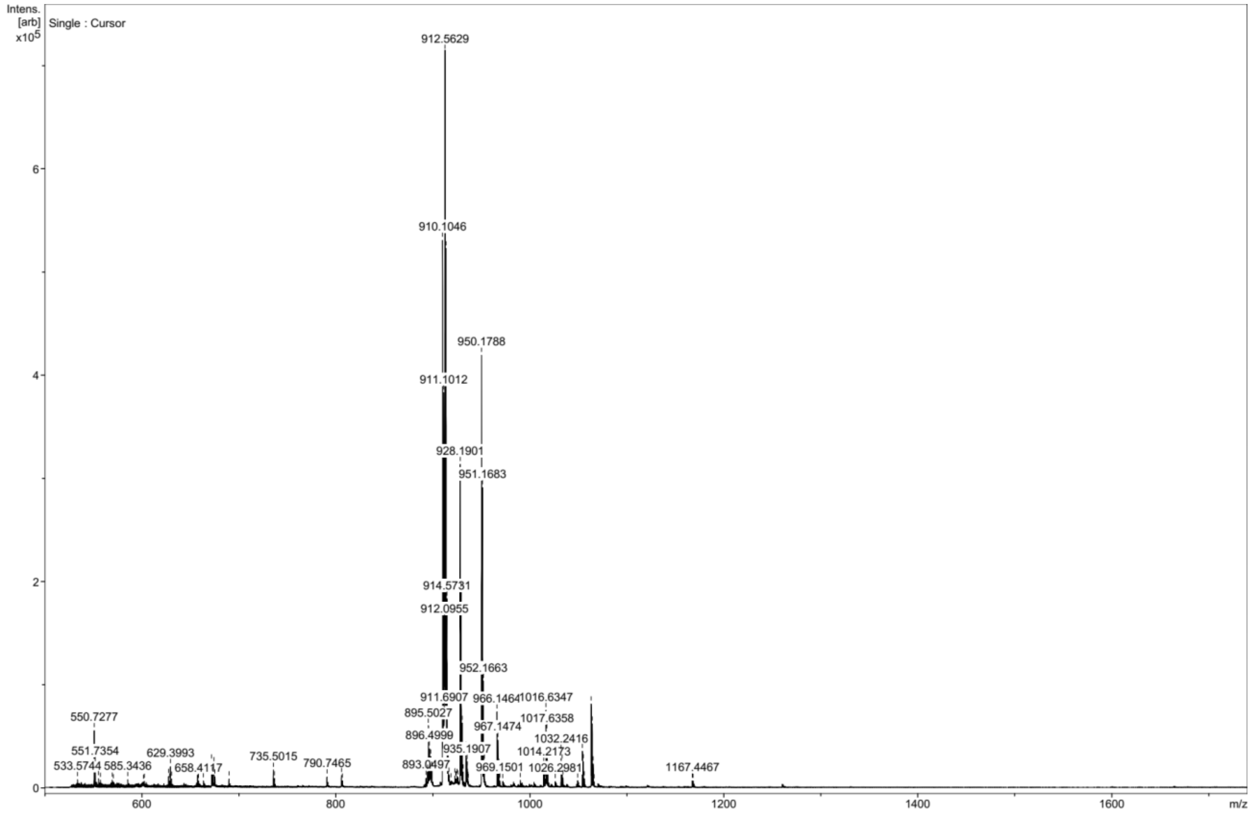

Intens. [arb] x10^5
Single : Cursor
912.5629
910.1046
950.1788
911.1012
928.1901
951.1683
914.5731
912.0955
952.1663
911.6907
966.1464
1016.6347
895.5027
1017.6358
967.1474
896.4999
1032.2416
935.1907
1014.2173
550.7277
551.7354
629.3993
735.5015
533.5744
585.3436
658.4117
790.7465
893.0497
969.1501
1026.2981
1167.4467
m/z

Compound 6

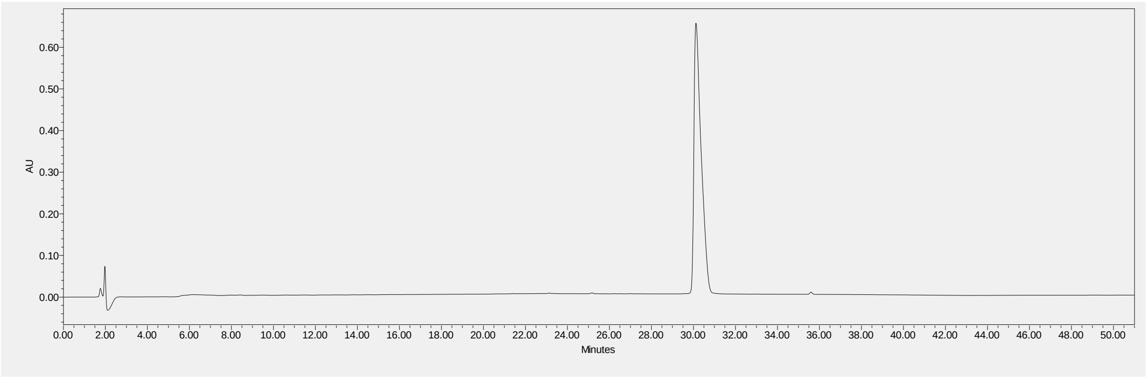

AU
Minutes

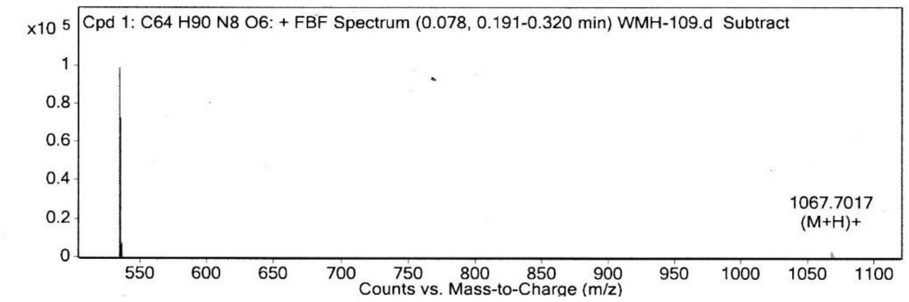

Compound 7

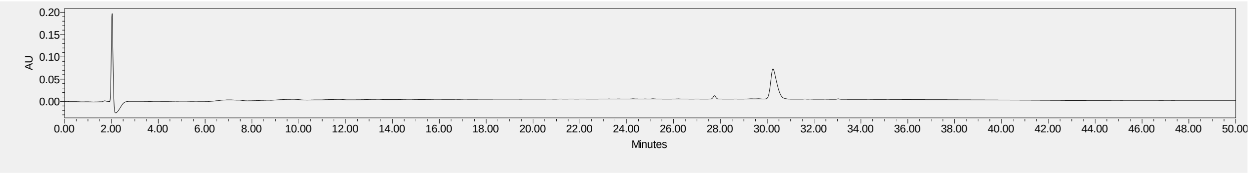

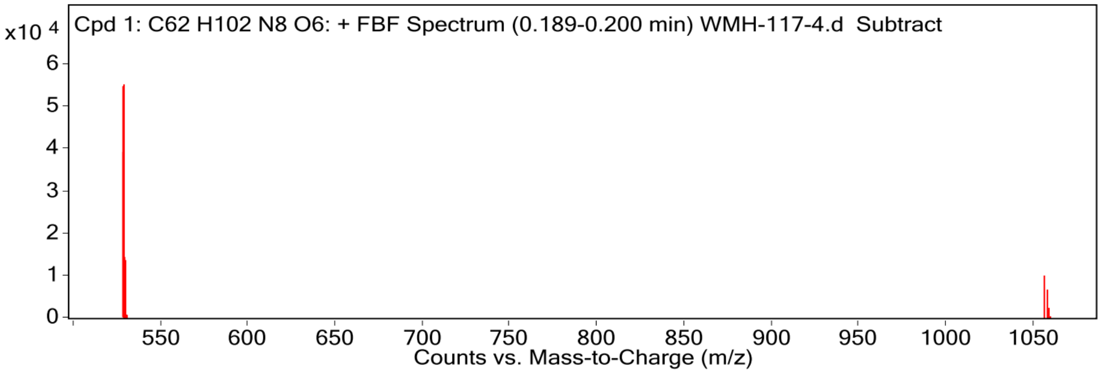

MS Spectrum Peak List

m/z	z	Abund	Formula	Ion
528.4021	2	55460.61	C62H104N8O6	(M+2H)+2
528.9037	2	38861.81	C62H104N8O6	(M+2H)+2
529.4046	2	13896.05	C62H104N8O6	(M+2H)+2
529.9064	2	3295.72	C62H104N8O6	(M+2H)+2
530.4075	2	870.34	C62H104N8O6	(M+2H)+2
1055.7969	1	10433.45	C62H103N8O6	(M+H)+
1056.7993	1	7029.73	C62H103N8O6	(M+H)+
1057.801	1	2792.69	C62H103N8O6	(M+H)+
1058.8021	1	565.24	C62H103N8O6	(M+H)+
1059.8093	1	72.43	C62H103N8O6	(M+H)+

Compound 8

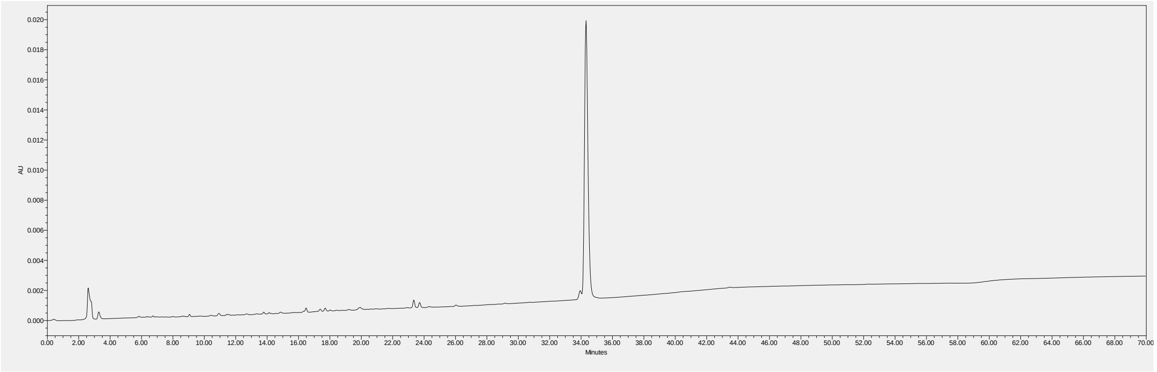
AU
Minutes

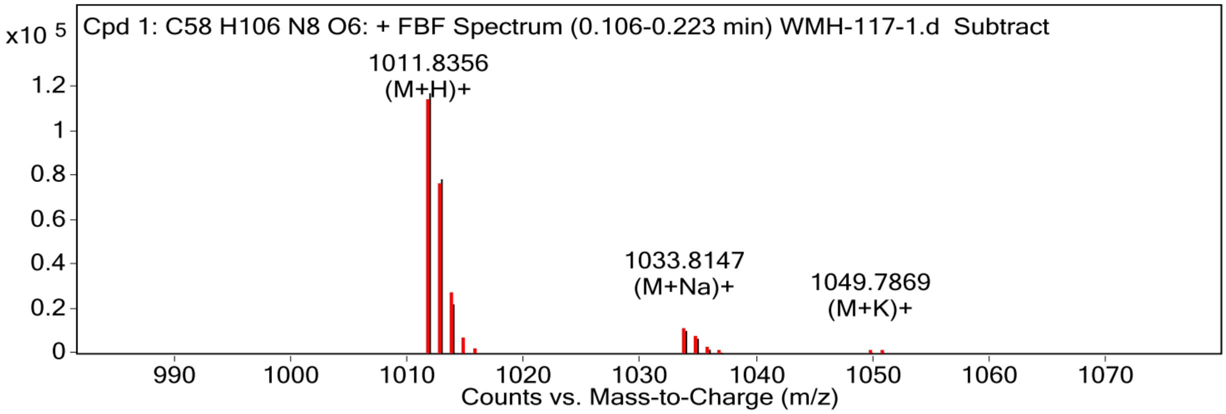
x10 5
Cpd 1: C58 H106 N8 O6: + FBF Spectrum (0.106-0.223 min) WMH-117-1.d Subtract
1011.8356
(M+H)+
1033.8147
(M+Na)+
1049.7869
(M+K)+
1.2
1
0.8
0.6
0.4
0.2
0
990
1000
1010
1020
1030
1040
1050
1060
1070
Counts vs. Mass-to-Charge (m/z)

Compound 9

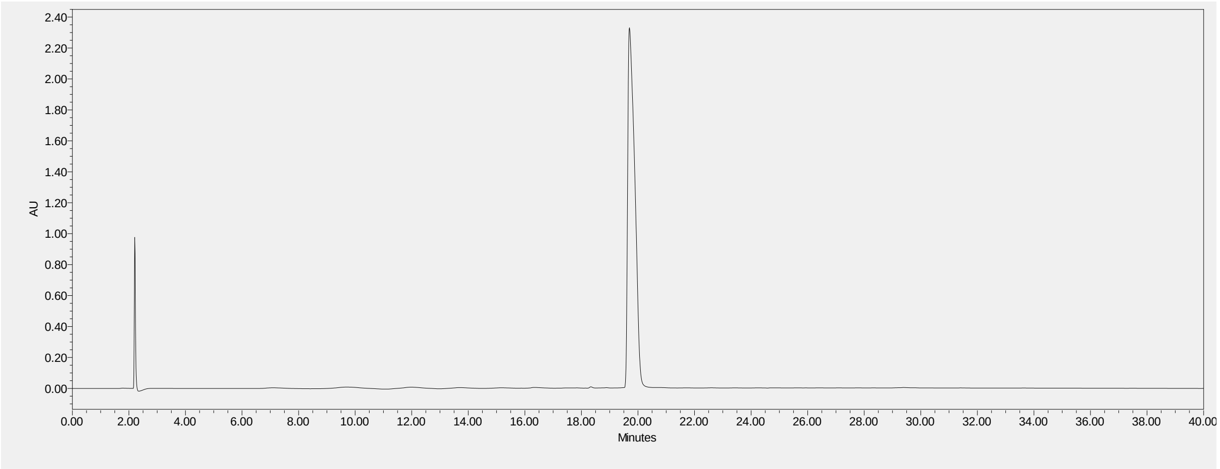
AU
2.40
2.20
2.00
1.80
1.60
1.40
1.20
1.00
0.80
0.60
0.40
0.20
0.00
0.00
2.00
4.00
6.00
8.00
10.00
12.00
14.00
16.00
18.00
20.00
22.00
24.00
26.00
28.00
30.00
32.00
34.00
36.00
38.00
40.00
Minutes

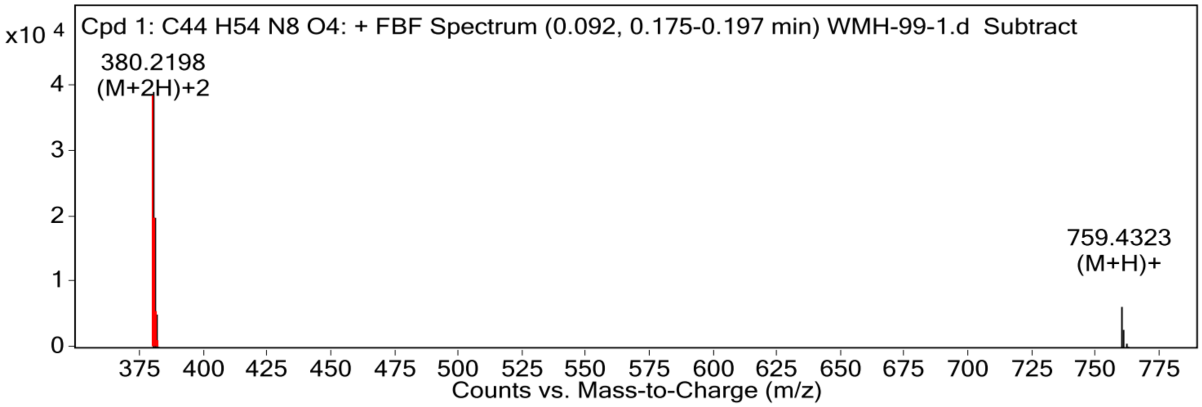

Compound 10

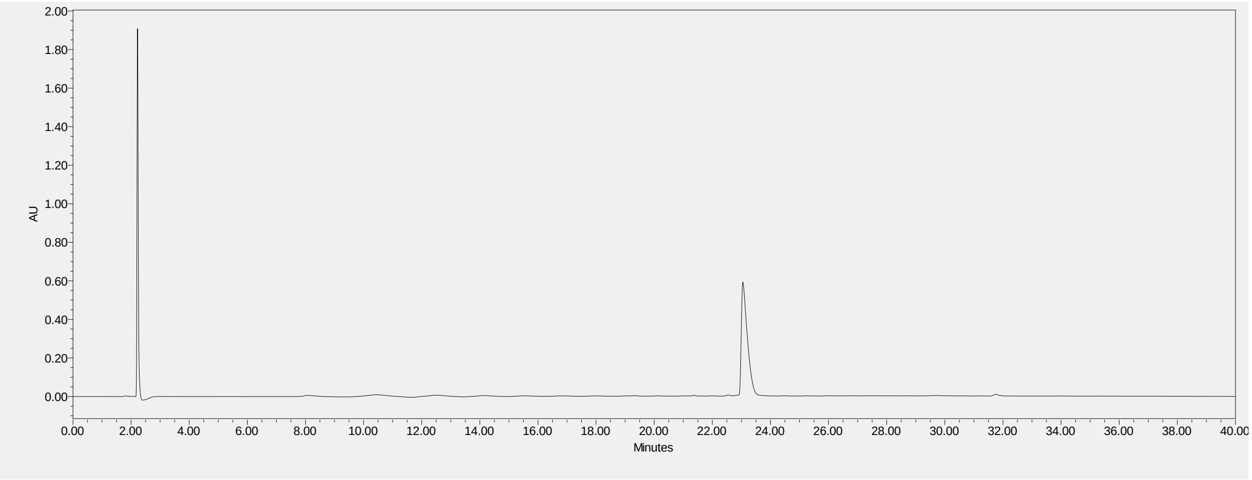

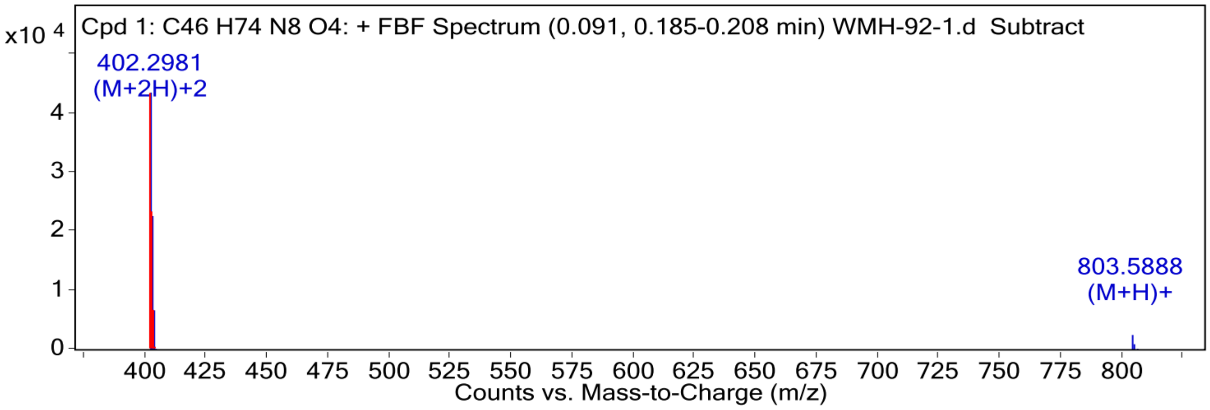

Compound 11

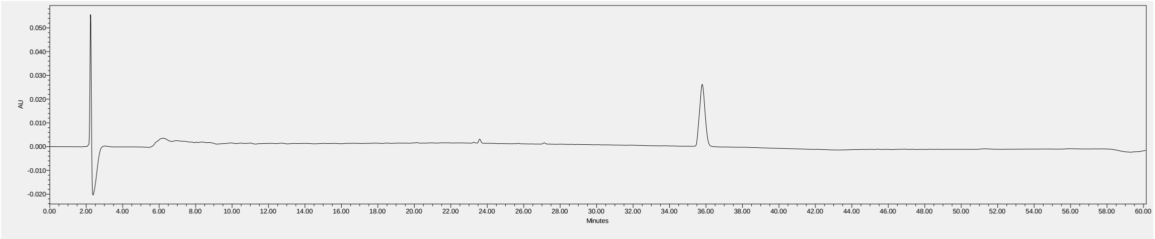

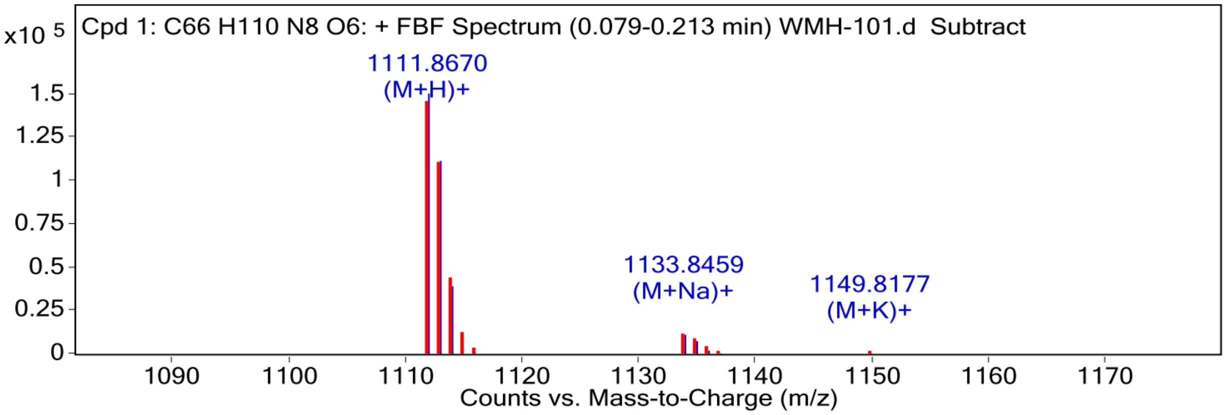

x10 5
Cpd 1: C66 H110 N8 O6: + FBF Spectrum (0.079-0.213 min) WMH-101.d Subtract
1111.8670
(M+H)+
1133.8459
(M+Na)+
1149.8177
(M+K)+
Counts vs. Mass-to-Charge (m/z)

Compound 12

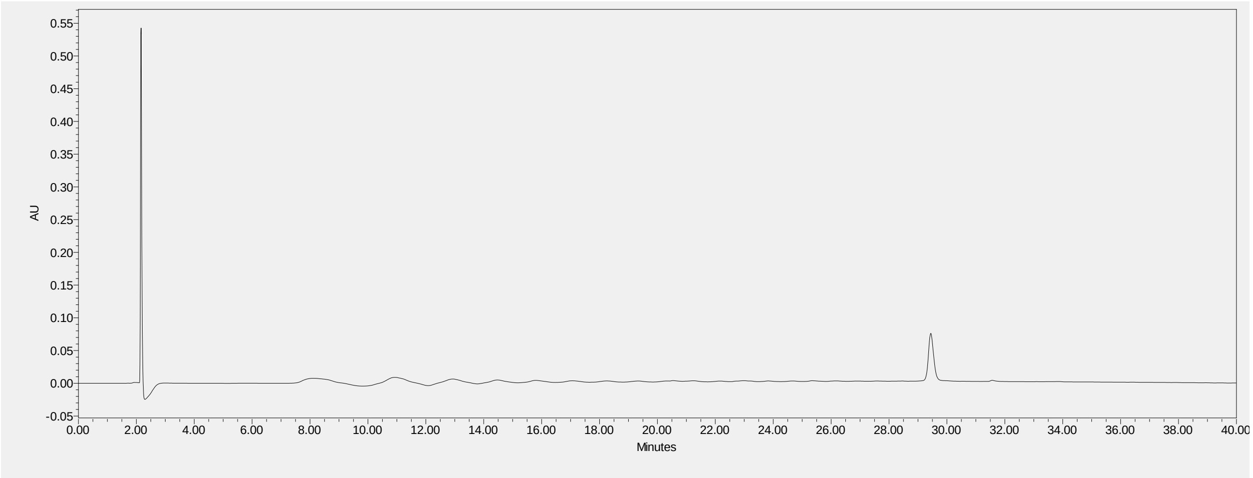

AU
Minutes

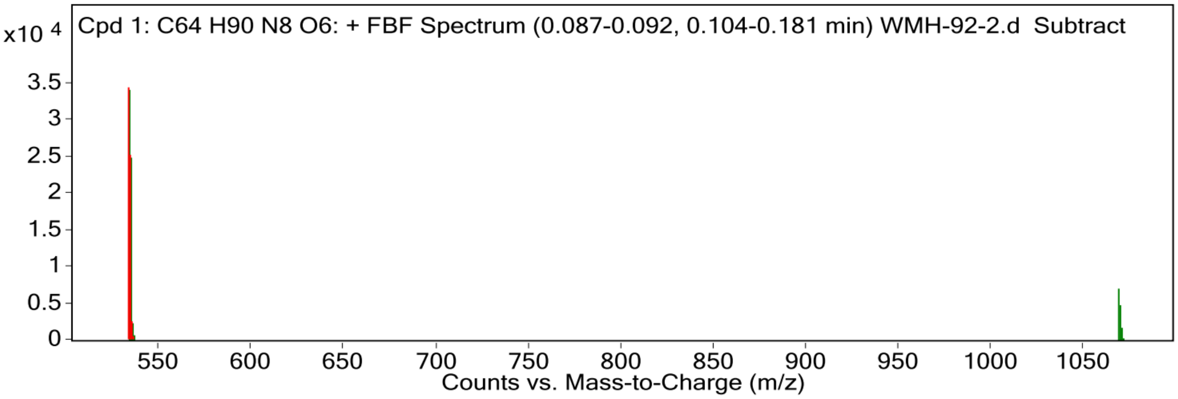

MS Spectrum Peak List

m/z	z	Abund	Formula	Ion
534.355	2	34149.53	C64H92N8O6	(M+2H)+2
534.8564	2	24870.26	C64H92N8O6	(M+2H)+2
535.3578	2	9512.24	C64H92N8O6	(M+2H)+2
535.8598	2	2544.69	C64H92N8O6	(M+2H)+2
536.3635	2	750.05	C64H92N8O6	(M+2H)+2
1067.703	1	7232.37	C64H91N8O6	(M+H)+
1068.7055	1	4829.71	C64H91N8O6	(M+H)+
1069.7082	1	1799.24	C64H91N8O6	(M+H)+
1070.7105	1	390.43	C64H91N8O6	(M+H)+
1071.7118	1	66.57	C64H91N8O6	(M+H)+

Compound 13

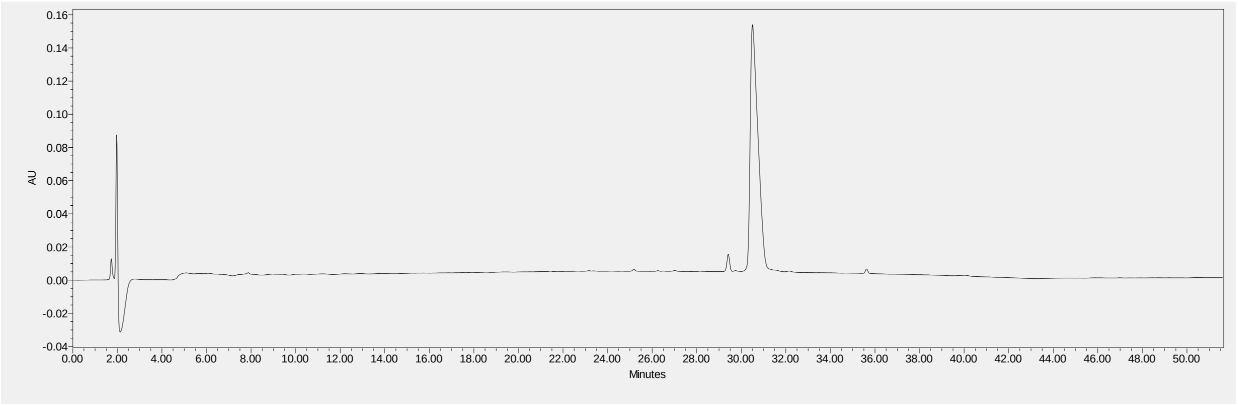

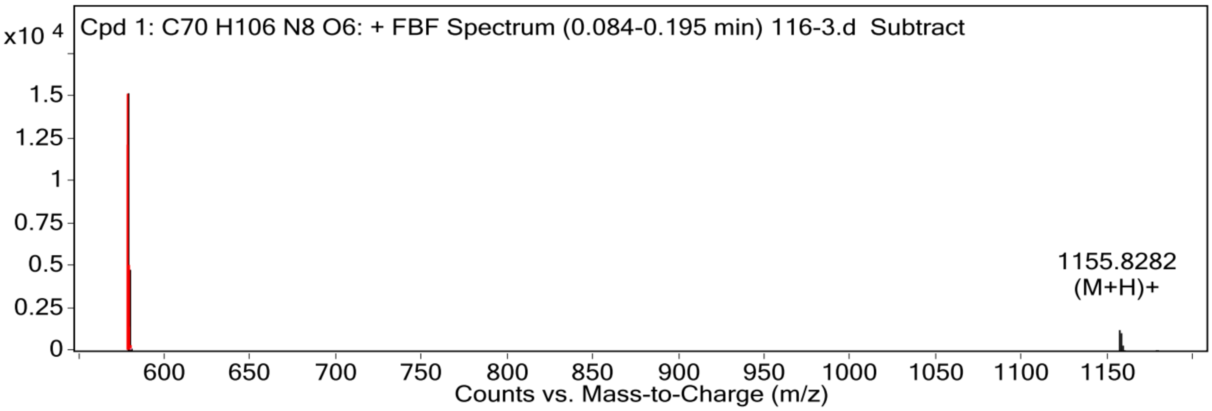

Compound 14

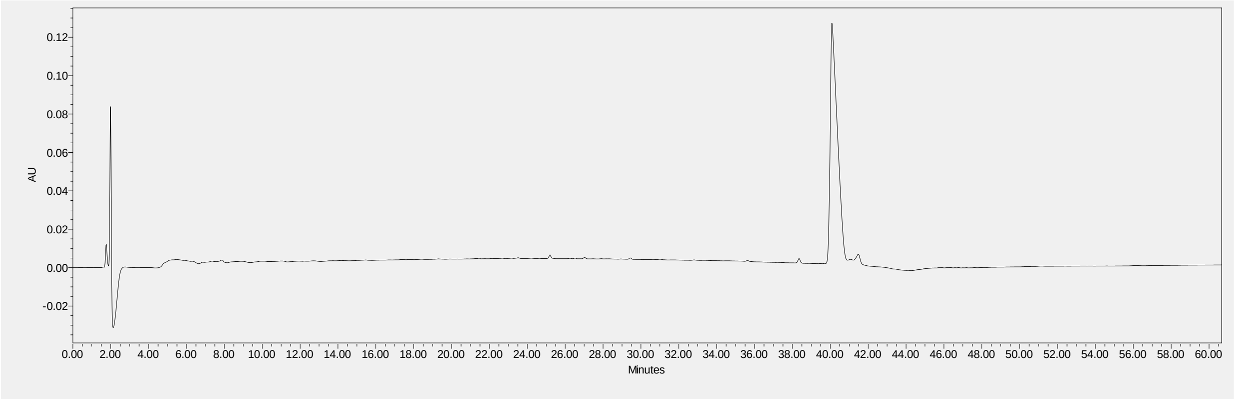

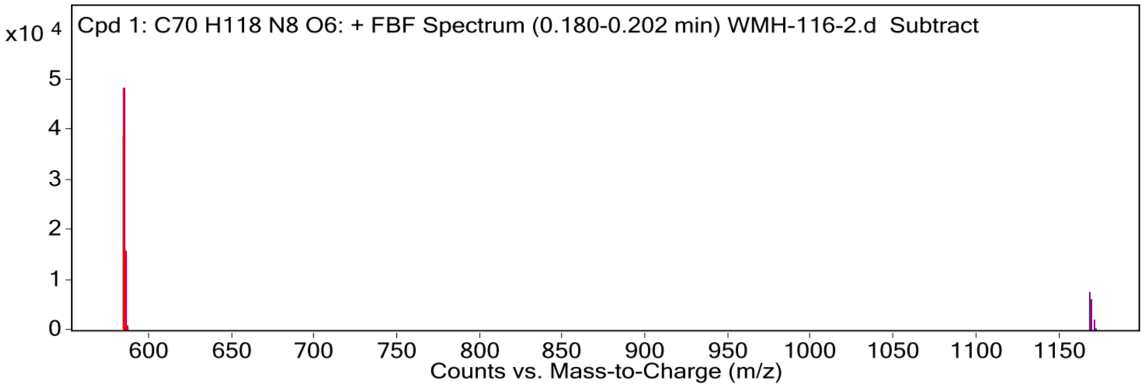

MS Spectrum Peak List

m/z	z	Abund	Formula	Ion
584.4647	2	48529.57	C70H120N8O6	(M+2H)+2
584.9662	2	38254.34	C70H120N8O6	(M+2H)+2
585.4673	2	15949.09	C70H120N8O6	(M+2H)+2
585.9697	2	4253.4	C70H120N8O6	(M+2H)+2
586.4716	2	1070.64	C70H120N8O6	(M+2H)+2
586.9726	2	213.65	C70H120N8O6	(M+2H)+2
1167.9215	1	7748.92	C70H119N8O6	(M+H)+
1168.924	1	6465.25	C70H119N8O6	(M+H)+
1169.9276	1	2452.9	C70H119N8O6	(M+H)+
1170.9304	1	635.12	C70H119N8O6	(M+H)+
